# Bioequivalence of a new coated 15 mg primaquine formulation for malaria elimination

**DOI:** 10.1186/s12936-024-04947-6

**Published:** 2024-06-05

**Authors:** Julie Nguyen Ngoc Pouplin, Thoopmanee Kaendiao, Bilal Ahmad Rahimi, Mayur Soni, Hensi Basopia, Darshana Shah, Jitendra Patil, Vyom Dholakia, Yash Suthar, Joel Tarning, Mavuto Mukaka, Walter R. Taylor

**Affiliations:** 1 Réseau Médicaments et Développement, 21Bis Avenue du Commandant l’Herminier, 44600 Saint-Nazaire, France; 2 Mahidol Oxford Tropical Medicine Clinical Research Unit, Mahidol University, Bangkok, Thailand; 3 Department of Paediatrics, Faculty of Medicine, Kandahar University, Kandahar, Afghanistan; 4 Cliantha Research Limited, Cliantha Corporate, Ahmedabad, Gujarat India; 5 Centre for Tropical Medicine and Global Health, Nuffield Department of Medicine, University of Oxford, Oxford, UK

**Keywords:** Primaquine, Bioequivalence, Malaria, Pharmacokinetics

## Abstract

**Background:**

With only one 15 mg primaquine tablet registered by a stringent regulatory authority and marketed, more quality-assured primaquine is needed to meet the demands of malaria elimination.

**Methods:**

A classic, two sequence, crossover study, with a 10-day wash out period, of 15 mg of IPCA-produced test primaquine tablets and 15 mg of Sanofi reference primaquine tablets was conducted. Healthy volunteers, aged 18–45 years, without glucose-6-phosphate dehydrogenase deficiency, a baseline haemoglobin ≥ 11 g/dL, creatinine clearance ≥ 70 mL/min/1.73 ms, and body mass index of 18.5–30 kg/m^2^ were randomized to either test or reference primaquine, administered on an empty stomach with 240 mL of water. Plasma primaquine and carboxyprimaquine concentrations were measured at baseline, then 0.25, 0.5, 0.75, 1.0, 1.25, 1.5, 1.75, 2.0, 2.333, 2.667, 3.0, 3.5, 4.0, 4.5, 5.0, 5.5, 6.0, 8.0, 10.0, 12.0, 16.0, 24.0, 36.0, 48.0 and 72.0 h by liquid chromatography coupled to tandem mass spectrometry. Primaquine pharmacokinetic profiles were evaluated by non-compartmental analysis and bioequivalence concluded if the 90% confidence intervals (CI) of geometric mean (GM) ratios of test vs. reference formulation for the peak concentrations (*C*_max_) and area under the drug concentration–time (AUC_0–t_) were within 80.00 to 125.00%.

**Results:**

47 of 50 volunteers, median age 33 years, completed both dosing rounds and were included in the bioequivalence analysis. For primaquine, GM *C*_max_ values for test and reference formulations were 62.12 vs. 59.63 ng/mL, resulting in a GM ratio (90% CI) of 104.17% (96.92–111.96%); the corresponding GM AUC_0–t_ values were 596.56 vs. 564.09 ngxh/mL, for a GM ratio of 105.76% (99.76–112.08%). Intra-subject coefficient of variation was 20.99% for *C*_max_ and 16.83% for AUC_0–t_. Median clearances and volumes of distribution were similar between the test and reference products: 24.6 vs. 25.2 L/h, 189.4 vs. 191.0 L, whilst the median half-lives were the same, 5.2 h.

**Conclusion:**

IPCA primaquine was bioequivalent to the Sanofi primaquine. This opens the door to prequalification, registration in malaria endemic countries, and programmatic use for malaria elimination.

*Trial registration* The trial registration reference is ISRCTN 54640699

**Supplementary Information:**

The online version contains supplementary material available at 10.1186/s12936-024-04947-6.

## Background

Primaquine (PQ), an 8-aminoquinoline anti-malarial drug, has taken on increasing importance for malaria elimination in recent years. It has long been recommended for radical cure to prevent relapses of *Plasmodium vivax* and *Plasmodium ovale*, and, since 2012, single low dose PQ (target dose 0.25 mg/kg body weight; 15 mg in adults) has been recommended for blocking the transmission of, especially, artemisinin-resistant *Plasmodium falciparum* in low transmission areas [1], replacing the previous dose of 0.75 mg/kg [2, 3]. The PQ doses in adults for radical cure are 15 mg × 14 days or 30 mg × 7 or 14 days, and 45 mg weekly for 8 weeks in glucose-6-phosphate dehydrogenase deficient (G6PDd) patients [4].

Published data on the pharmacokinetics (PK) of orally administered PQ show good absorption to reach a maximum plasma concentration (*C*_max_) in one to three hours, followed by a decline with a mean terminal elimination half-life of approximately 4 h [5, 6]. PQ is primarily metabolized by monoamine oxidase A, which produces the metabolically inactive carboxyPQ [7]. The pathway responsible for PQ’s active, oxidative metabolites is the highly polymorphic cytochrome (CYP) P450 2D6 with 4 categories of metabolizer status: poor, intermediate, normal and ultrarapid [8]. As the active metabolites generated via this pathway are unstable and difficult to measure, data on their PK profiles and pharmacodynamic relationships are currently unknown [9]. Nevertheless, the active metabolites are responsible for the gametocytocidal [10] and antirelapse [11] activities of PQ and its key toxicity of dose-dependent, acute haemolysis in G6PDd individuals [12, 13].

PQ is characterized by dose proportional PK, i.e., the *C*_max_ and exposure [area under the concentration–time curve, (AUC)] roughly double with a doubling of the administered dose [6, 14]. Following 15, 30 and 45 mg of PQ to the same 5 healthy adult volunteers, the measured mean *C*_max_ values were 53, 104, and 176 ng/mL, respectively, and the corresponding mean AUC_0–24_ values were 0.5, 1.0, and 1.6 µg × h/mL [6]. Consistent findings are also reported by Khan et al. [14] who documented a doubling of *C*_max_ and AUC_0–24_ following the administration of 15–30 or 22.5–45 mg of (+)-*S* enantiomer and racemic PQ, as well as Daher et al. [15] who recorded geometric mean *C*_max_ values in Brazilian adults of 65.33 and 21.73 ng/mL, following 15 and 5 mg tablets, respectively. Food enhances PQ absorption and, in one study, two buttered bread rolls, providing 28 g of fat, increased the *C*_max_ by 26% and AUC_0–∞_ by 14% in 20 healthy Vietnamese males (n = 10) and females (n = 10) [16].

Currently, there is only one 15 mg PQ tablet that is registered by a stringent regulatory authority (SRA) and marketed; this is the US FDA-registered, 15 mg, coated, unflavoured tablet produced by Sanofi. In December 2023, the World Health Organization (WHO) has prequalified a 15 mg, coated, unflavoured tablet, produced by Macleods in India. A 7.5 mg coated, unflavoured tablet is also registered at the Cypriot Ministry of Health Pharmaceutical Services and marketed by Remedica, a local manufacturer [17]. The Global Fund, set up to help control programmes meet the high cost of drugs for TB, Malaria and HIV, only bulk buys SRA-approved or WHO-prequalified anti-malarials. The current PQ-producing capacity is insufficient to meet global needs, not to mention the lack of paediatric-friendly PQ formulations, and, therefore, more manufacturers are needed to enter the market with inexpensive, quality-assured PQ dosage forms. One requirement to prequalifying adult strength PQ tablets is to conduct a bioequivalence (BE) study against the Sanofi reference. Herein, the results of a BE study comparing a new 15 mg PQ generic, produced by IPCA in India, with the Sanofi 15 mg product are reported.

## Methods

### Ethical and other approvals

The study (protocol ref: C1B00842–BE-PRIM) was approved by the: (i) Ibiome Independent Ethics Committee (India) on the 28th of November 2022, and (ii) the Oxford University Tropical Ethics Committee (OxTREC Reference: 40-21) on the 2nd of December 2022. The study protocol was also reviewed by the Prequalification team at the WHO in Geneva, in accordance with the standard practice for studies intended for WHO prequalification.

### Study design and site

This was an open label, randomized, two-period, two treatment, two-sequence, crossover, balanced single dose, oral bioavailability study in healthy adults under fasting conditions; it was conducted from the 16th to the 30th of December 2022 by Cliantha, an independent clinical research organization, at their main clinical site in Ahmedabad, India.

### Study participants

Potential volunteers were selected from a pool of registered individuals held by Cliantha; all were Indian nationals. They were invited to attend an information session on this and other studies and given the opportunity to select the study they wished to join. Those expressing an interested for this BE study then went through the informed consent process and were assessed by history, physical examination, and laboratory testing. As a precaution against not meeting the sample size (e.g. pulling out at the last minute), more than the required number of participants were selected to attend dosing in the first round.

Male and non-pregnant, non-lactating volunteers who gave informed consent and met all of the following inclusion criteria were included in the study: (i) aged 18 to 45 years old, (ii) females of childbearing potential had to be on reliable contraception, (iii) body mass index (BMI) of 18.5 to 30.0 kg/m^2^, (iv) non-smokers and non-tobacco user for ≥ 1 year, (v) judged healthy by the examining physician, (vi) normal chest X-ray, ECG and normal laboratory results (full blood count, routine biochemistry, urine dipstick analysis, HIV 1 & 2, VDRL, hepatitis B surface antigen, hepatitis C antibody, qualitative screening for glucose-6-phosphate dehydrogenase deficiency), and (vii) a negative urine screen for alcohol and recreational drugs (e.g. marijuana, amphetamine, barbiturates, cocaine); urine screening was also performed on the day of each check-in period.

Volunteers were excluded if they met any of the following criteria: (i) allergic to PQ, (ii) presence of a significant disease or clinically significant abnormal findings during screening, (iii) known to have a significant disease of any physiological system e.g. diabetes mellitus, TB, psychosis, (iv) use of any hormone replacement therapy or depot injection or implant of any drug within 3 months of first dose, (v) used CYP enzyme inhibitors or inducers within the previous 30 days, (vi) drug/alcohol dependence or moderate alcohol use, (vii) difficulty with donating blood/vein accessibility or intolerant of venepuncture, (viii) received a known investigational drug within seven elimination half-lives of the administered drug prior to the first dose of study medication, (ix) donated blood or blood loss within 90 days, (x) difficulty swallowing, (xi) food allergy/intolerance or on a restricted diet, (xii) use of any prescribed medications ≤ 14 days and/or over the counter products e.g. vitamins, herbals ≤ 7 days, (xiii) consumed grapefruit (a potent CYP 3A4 inhibitor) or grapefruit products ≤ 7 days, (xiv) ingestion of any caffeine or xanthine products (i.e. coffee, tea, chocolate, and caffeine-containing sodas, colas), recreational drugs, alcohol or other alcohol containing products within 48 h, and (xv) family or personal history of haemolytic anaemia.

### COVID-19 precautions

All participants were screened prior to study enrolment and before each check-in for COVID-19 by symptoms, vaccine and contact history, physical examination, oxygen saturation (SpO_2_), and thermal scanning. Those with suspected COVID-19 were referred for testing. For enrolled volunteers, the following measures were put in place: (i) provision of hand sanitizer, soap, and facemasks, (ii) information posters/instructions were placed at all working areas, and (iii) social distancing was followed at all times by the staff and volunteers during the entire period of clinical conduct.

### Test and reference products

The test PQ tablet was produced by IPCA Laboratories, Athal, India, and contained 15 mg of PQ base (26.3 mg PQ diphosphate). The batch number is HTZ0220019 and has an expiry date of September 2024 (2y shelf life). The reference PQ, 15 mg base (26.3 mg PQ diphosphate), was manufactured by Sanofi-aventis, New Jersey, USA; the batch number is 8,125,520 with an expiry date of August 2024.

### Randomization and drug administration

Volunteers were randomized to receive one of the two PQ formulations in period 1, and the opposite formulation in period 2, following a computer-generated randomization schedule (SAS statistical software v. 9.4, SAS Institute Inc., USA). The site principal investigator and pharmacist ensured compliance to the randomization schedule.

After an overnight fast (≥ 10 h), a single 15 mg PQ tablet was administered by a research team member to each volunteer in the sitting position with ~ 240 mL of water. PQ was swallowed whole and not chewed, crushed or divided. After dosing, the mouth and hands were checked to confirm PQ had been taken.

For 4 h after the first dose, volunteers were required to remain sitting up (limited movement allowed), to minimize the inter-individual variation in PQ absorption due to changes in posture; compared to being supine, sitting up is associated with increased drug absorption, especially for drugs that have a significant first pass effect and when taken with water on an empty stomach [18]. After this, a standard meal was served and, later, snacks/food were given, according to a defined schedule, and were the same for each volunteer. No water was allowed to be consumed 2 h post first dosing. A number of additional restrictions included no consumption of coffee, tea, chocolate, colas and alcohol 48 h prior to dosing, and no grapefruit or grapefruit products 7 days before dosing.

### Clinical evaluations

Pulse and blood pressure were measured at 2, 6, and 10 h post dose and prior to discharge. Volunteers were asked about symptoms at these times and 16 and 24 h after dosing to detect adverse events. Volunteers were discharged after 24 h in each study period. At check-in for period 2, patients underwent a physical examination and a repeat full blood count. Adverse events were graded, according to the MedDRA system, and laboratory normal ranges were based on data in Indian individuals (Additional file 1: Table S1). Methaemoglobinaemia was not measured.

### Pharmacokinetic sampling and bioanalysis

Whole blood samples were collected via an intravenous canula into EDTA tubes at pre-dose (0.0 h) and at 0.25, 0.5, 0.75, 1.0, 1.25, 1.5, 1.75, 2.0, 2.333, 2.667, 3.0, 3.5, 4.0, 4.5, 5.0, 5.5, 6.0, 8.0, 10.0, 12.0, 16.0, 24.0, 36.0, 48.0 and 72.0 h after the administration of each dose.

The cannula was kept patent by injecting 0.5 mL of normal saline solution after each sample; subsequent blood samples were collected after discarding the first 0.5 mL of blood. After collection, the blood samples were placed in an ice bath, transferred to a refrigerated centrifuge within 45 min of collection and spun at 4000G at 4 °C for 10 min. Two aliquots of 1.2 mL of plasma were transferred to labelled polypropylene tubes: aliquot 1 (primary aliquot) and aliquot 2 (secondary aliquot), and stabilized with 50 µL of 1.0 M sodium fluoride solution (w/v)/1.0 mL of plasma in a wet ice bath before storage in a − 70 °C ± 10 °C freezer.

Plasma PQ and carboxyPQ were measured by liquid chromatography coupled to tandem mass spectrometry using the API 5500 spectrometer (AB Sciex, MA, CA, USA) and the Kinetex Biphenyl 100A° 5 μm 100 × 4.6 mm (00D-4627-E0) HPLC column (Torrance, CA, USA). The lower limits of quantification were 0.50 ng/mL for PQ and 5.00 ng/mL for carboxyPQ; sample values below these limits of quantification were considered as zero in the PK analysis.

### Sample size calculation, determining bioequivalence and power

The sample size was calculated following US FDA and EMA guidance for determining the bioequivalence of primaquine, the primary study endpoint. The primary PK parameters to determine bioequivalence were the natural logarithm (Ln) of individual parameter estimates of *C*_max_ and AUC_0–t_ of PQ after receiving the test and reference PQ formulations, where t, 48 h, is the time of the last sample used in the analysis. The geometric mean ratio and its corresponding 90% confidence interval of the test vs. reference for both *C*_max_ and AUC_0–t_ must fall within 80.00 to 125.00% for bioequivalence to be concluded.

Previous PQ interaction studies in healthy volunteers showed that the within-subject coefficient of variation of exposure parameters was < 21% [19, 20]. For this study, we assumed a CV of 25% to increase power, and a maximum true difference between the test and reference formulations of 5% of the exposure and peak concentration. For a power of 95%, a two one-sided alpha of 0.05, the sample size was 45 evaluable volunteers. Allowing for loss to follow up, 50 volunteers were recruited. The sample size calculations were performed in SAS software (Version: 9.4; SAS Institute Inc, USA).

The Ln transformed PQ *C*_max_ and AUC_0–t_ data were evaluated by analysis of variance (PROC GLM in SAS) for differences due to treatment, period, sequence and subject (sequence) as fixed effects. Treatment and period were tested using the mean square error and sequence was tested using subject (sequence) as the error term at 5% level of significance. The intra-subject variability and power for the Ln-transformed PQ *C*_max_ and AUC_0–t_ were computed using the two one-sided test method.

### Pharmacokinetic analysis

The PK analysis was performed using SAS statistical software 9.4 using non-compartmental analysis and included all volunteers who completed both study periods with sufficient data to conduct the bioequivalence determination. In the event, no participant had missing data, except one with a missing sample at 72 h so his AUC_0–t_ for carboxyPQ could not be estimated. The arithmetic mean, geometric mean, standard deviation, median, maximum and minimum for all pertinent PK parameters were calculated. The individual concentration–time data were analysed to derive the *C*_max_, *T*_max_ (time to maximum drug concentration), AUC_0–t_ (the last time points were 48 and 72 h for PQ and carboxyPQ, respectively), AUC_0–∞_, AUC__%Extrap_obs_, ke (terminal elimination rate constant), clearance (CL/*F*), volume of distribution (Vz/*F*), and t_1/2_ (terminal elimination half-life).

The *C*_max_ and *T*_max_ were taken directly from the observed data. The AUC up to the last measured drug concentration (AUC_0–t_) was calculated using the cubic spline method for ascending concentrations and the logarithmic cubic spline method for descending concentrations. The ke was estimated by the log-linear best-fit regression of the observed concentrations in the terminal elimination phase. Drug exposure was extrapolated from the last observed concentration to time infinity by *C*last/ke for each subject to compute the total drug exposure (AUC_0–∞_). The t_1/2_ was estimated by Ln2/ke. The apparent volume of distribution and oral clearance were calculated according to Eqs. 1 and 2, respectively.

For the calculation of the carboxyPQ clearance and volume of distribution, complete in vivo conversion of PQ into carboxyPQ was assumed and the equivalent carboxyPQ dose (Eq. 3) calculated, using the molecular weights of PQ (259.347 g/mol) and carboxyPQ (274.32 g/mol).

1 $$\frac{{{\text{CL}}}}{F} = \frac{{{\text{Dose}}}}{{AUC_{0 - \infty } }},$$

2 $$\frac{V}{F} = \frac{{CL \times t_{1/2} }}{Ln\left( 2 \right)},$$

3 $${\text{CarboxyPQ}}\;{\text{dose}} = \frac{PQ\;dose \times 274.32}{{259.347}}.$$

## Results

### Demographics

**Fig. 1:**
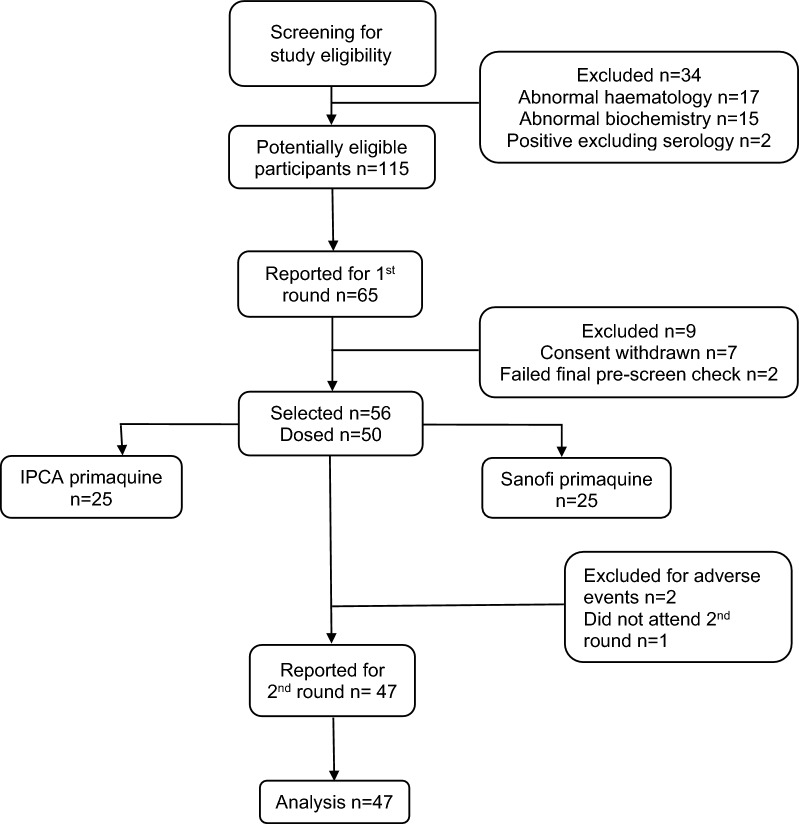
Trial profile

After the introductory talk on studies, 149 potential participants were screened for study eligibility; all were males (no females expressed an interest in joining this BE study), of whom 34 failed screening. From the pool of 115 potential participants, 65 agreed to attend check in and, of these, 56 were enrolled and 50 were dosed (Fig. 1). As per Cliantha’s standard procedures, 6 extra volunteers participated in period 1 of the study to guard against no show volunteers but only 50 were dosed with PQ. Of these 50, 1 volunteer did not attend in period 2 and two were discontinued from the study because of adverse events that developed after period 1. By study end, 47 volunteers with paired data were analysed for bioequivalence. The 50 male volunteers had a median age of 33 years, median BMI of 23.41 kg/m^2^, and median laboratory values that were all within their normal ranges (Table 1).

**Table 1:** Baseline characteristics

Parameter	Median (range)
Age (years)	33 (22 to 44)
Weight (kg)	64.9 (51 to 90)
Height (cm)	166.5 (151.5 to 180.5)
BMI (kg/m^2^)	23.1 (18.6 to 29.4)
Haematology	
Haemoglobin (g/dL)	14.9 (12.6 to 16.4)
Total WBC count (/µL)	7735 (4250 to 11,470)
Platelet count (/µL)	318,500 (170,000 to 488,000)
Biochemistry	
Sodium (mmol/L)	137.5 (133.5 to 139.6)
Potassium (mmol/L)	4.5 (3.6 to 5.2)
Creatinine (mg/dL)	0.865 (0.63 to 1.14)
Albumin (g/dL)	4.7 (4.1 to 5.2)
ALT (IU/L)	21.6 (8.9 to 61.8)
AST (IU/L)	21.5 (13.5 to 43.6)
Total bilirubin (mg/dL)	0.49 (0.21 to 1.06)

### Pharmacokinetic analysis and bioequivalence assessment

**Fig. 2:**
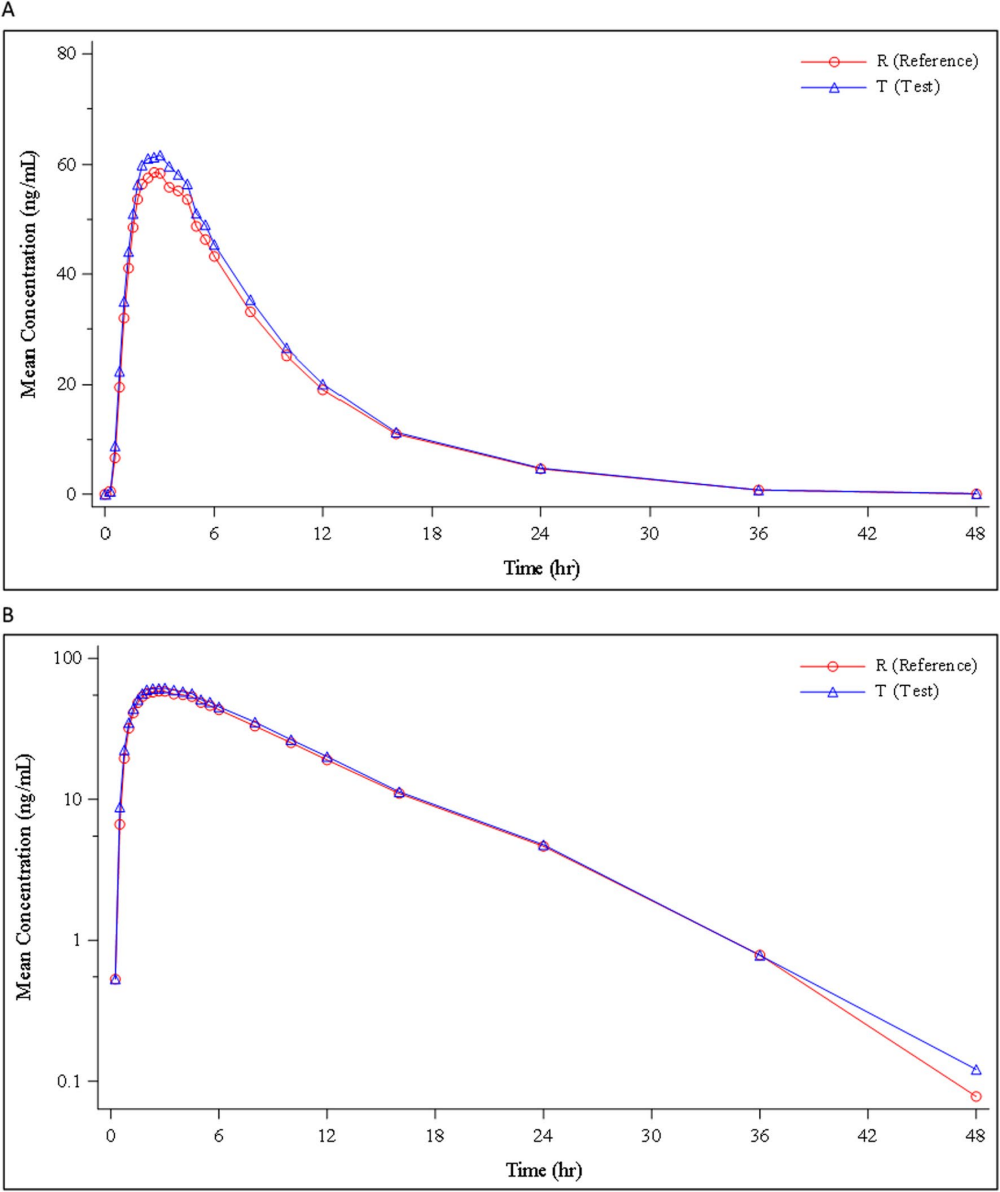
Mean concentration–time profile of measured primaquine in healthy volunteers (n = 47) receiving test (IPCA) and reference (Sanofi) primaquine formulations, shown both on an **A** arithmetic scale and **B** semi logarithmic scale

**Fig. 3:**
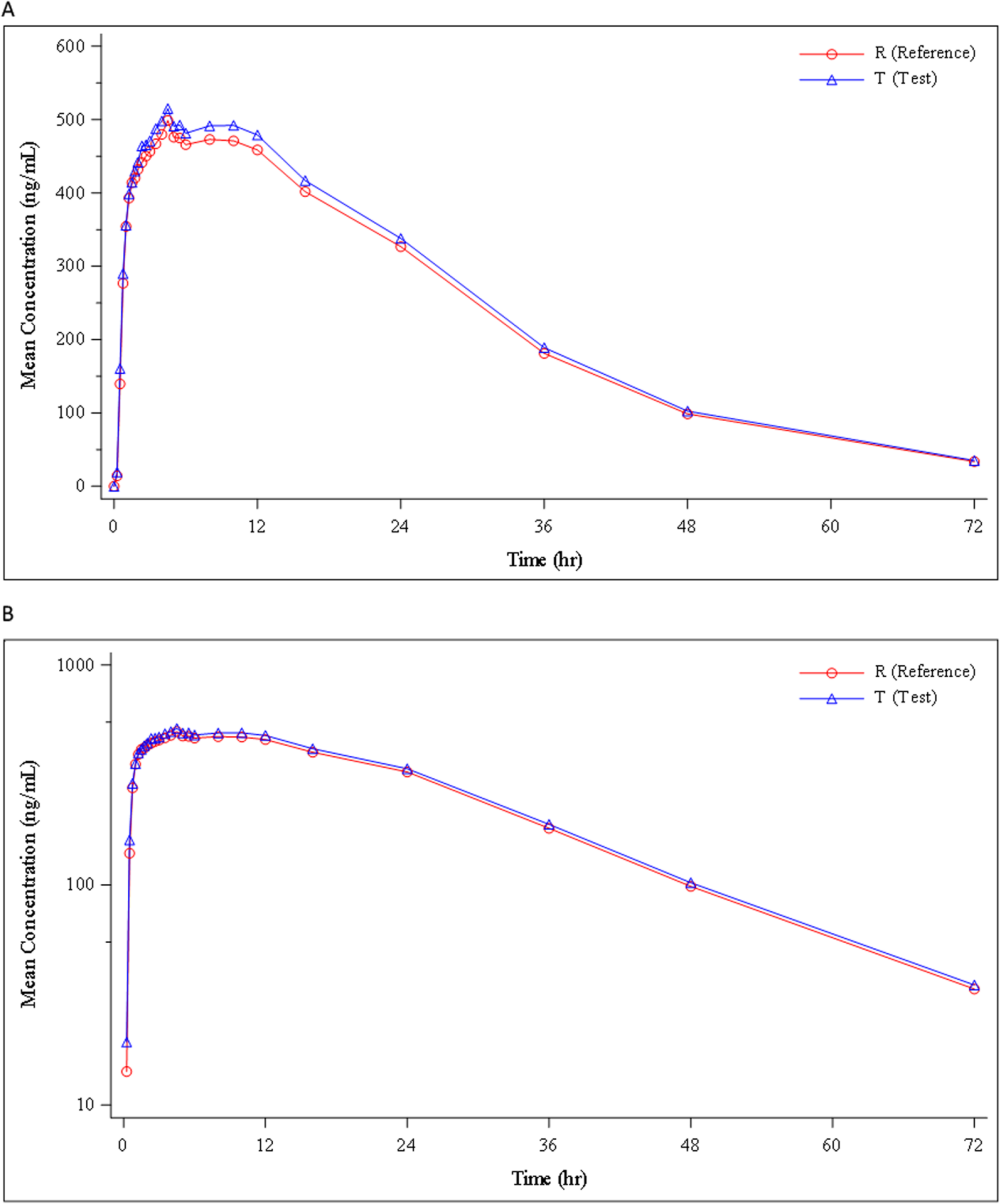
Mean concentration–time profile of measured carboxyprimaquine in healthy volunteers (n = 47) receiving test (IPCA) and reference (Sanofi) primaquine formulations, shown both on an **A** arithmetic scale and **B** semi logarithmic scale

Mean concentrations of PQ and carboxyPQ over time for both PQ formulations were virtually identical (Figs. 2 and 3). The GM values for the test and reference PQ are shown in Table 2 and show conclusively that the IPCA PQ product was bioequivalent to the Sanofi PQ formulation. All median PK parameters for test and reference PQ and carboxyPQ were also similar (Tables 3 and 4). Despite these findings, the ANOVA analysis showed that period was a significant explanatory factor (p = 0.0281) for the Ln AUC_0–t_ of PQ but this unexpected finding remains unexplained.

**Table 2:** The geometric means of the maximum concentrations and exposures for the test and reference primaquine formulation and the assessment of bioequivalence in 47 volunteers with paired data

Pharmacokinetic parameter	Geometric mean		Ratio (90% CI)
	Test	Reference	
*C*_max_ (ng/mL)	62.12	59.63	104.17 (96.92–111.96)
AUC_0–t_ (ng × h/mL)	596.56	564.09	105.76 (99.79–112.08)

*C*_max_: maximum drug concentration; AUC_0–t_: area under the drug concentration–time curve

**Table 3:** Pharmacokinetic data [median, range] for primaquine following 15 mg of IPCA (test) and Sanofi (reference) primaquine formulation in the 47 participants with paired data

Parameter	IPCA primaquine	Sanofi primaquine
*C*_max_ (ng/mL)	57.4 (31.5 to 169.5)	65.3 (28.4 to 116.9)
*T*_max_ (h)	2.7 (1.5 to 4.5)	2.7 (1.5 to 5.5)
CL/*F* (L/h)	24.6 (11.3 to 65.2)	25.2 (12.3 to 66.0)
Vz/*F* (L)	189.4 (76.6 to 343.5)	191.0 (104.3 to 437.7)
t_1/2_ (h)	5.2 (3.3 to 6.9)	5.2 (4.0 to 7.3)
AUC_0–t_ (ng × h/mL)	606.0 (227.1 to 1317.1)	577.1 (220.0 to 1210.7)
AUC_0–∞_ (ng × h/mL)	609.6 (230.2 to 1324.7)	596.0 (227.1 to 1217.5)

*C*_max_: maximum drug concentration; *T*_max_: time to maximum drug concentration; CL: total drug clearance from plasma; Vz: volume of distribution based on the terminal elimination phase; *F*: drug bioavailability; t_1/2_: terminal elimination half-life; AUC: area under the drug concentration–time curve

**Table 4:** Pharmacokinetic data [median, range] for carboxyprimaquine following 15 mg of IPCA (test) and Sanofi (reference) primaquine formulation in the 47 participants with paired data

Parameter	IPCA primaquine	Sanofi primaquine
*C*_max_ (ng/mL)	535.0 (326.4 to 758.1)	508.7 (297.1 to 719.6)
*T*_max_ (h)	4.5 (3.5 to 12.0)	4.5 (3.0 to 16.0)
CL/*F* (L/h)	0.84 (0.55 to 1.87)	0.84 (0.59 to 1.97)
Vz/*F* (L)	17.3 (12.6 to 28.3)	17.9 (12.5 to 31.5)
AUC_0–t_ (ng x h/mL)	17,203.5 (8022.7 to 24,581.2)	17,042.7 (7561.5 to 24,356.1)
AUC_0–∞_ (ng x h/mL)	17,945.5 (8002.7 to 27,071.0)	17,864.7 (7597.5 to 25,616.5)
t_1/2_ (h)	13.0 (6.9 to 24.2)	13.7 (6.4 to 19.9)

One subject had a missing sample at 72.0 h in period 2 and his AUC_0–t_ was not calculated

*C*_max_: maximum drug concentration; *T*_max_: time to maximum drug concentration; CL: total drug clearance from plasma; Vz: volume of distribution based on the terminal elimination phase; *F*: drug bioavailability; t_1/2_: terminal elimination half-life; AUC: area under the drug concentration–time curve

### Safety and tolerability

Both formulations of PQ were well tolerated and there were no reported clinical adverse events and no clinical or laboratory serious adverse events. A total of four, mild laboratory adverse events occurred in four volunteers; all were considered to be unlikely related to PQ.

One participant after receiving test PQ in the first period developed an exacerbation of eosinophilia and another participant in the first period developed leucocytosis after receiving reference PQ. At baseline, the test recipient had an absolute eosinophil count of 973/µL (8% of 12,170/µL) that rose to 2350/µL (25.3% of 9290/µL); this resolved spontaneously to 95/µL (1.1% of 8620/µL). The leucocytosis in the reference recipient was 13,510/µL from a baseline of 10,290/µL that fell to 11,630/µL on repeat testing.

After study end (i.e. both periods), one volunteer developed an asymptomatic increase in ALT of 101.7 IU/L from a baseline of 48.8 IU/L that fell to 72 IU/L on repeat testing; he received test followed by reference PQ. Another volunteer, also after study completion, developed a leucocytosis of 12,630/µL from a baseline of 10,350/µL; he received reference followed by test PQ.

## Discussion

This study shows that a new, generic, IPCA-produced form of 15 mg of PQ is bioequivalent to the reference 15 mg formulation produced by Sanofi and both were well tolerated. This positive result fulfils a key requirement for obtaining WHO prequalification of IPCA PQ.

It was purposefully set out to conduct a large study by opting for a high statistical power of 95% and inflating the intrasubject coefficient of variation. In the end, the IPCA PQ was bioequivalent with a statistical power of 99% and an intrasubject coefficient of variation of 21%, found also in Thai adult volunteers, was reconfirmed [20]. The bioanalysis used a validated method that Cliantha developed and the PK parameters obtained are consistent with those reported previously from a range of studies. The GM *C*_max_ of 62 ng/mL (≈ 66 ng/mL) is very close to the 65 ng/mL in Brazilian adults [15], broadly similar to the 53 ng/mL in 5 adult volunteers [6] and the 56 ng/mL in vivax-infected, Thai adults given 15 mg of PQ after chloroquine [21], as well as being around half of the median *C*_max_ reported in three studies of Thai adult volunteers given 30 mg of PQ: 122 [22], 128 [19] and 139 ng/mL [20].

The GM PQ exposure (AUC_0–t_) of a little under 600 ng × h/mL (≈ 635 ng × h/mL) was higher than the GM mean exposure (t was 24 h vs. our 48 h) of 563 ng × h/mL in the Brazilians and in 5 healthy volunteers (500 ng × h/mL [6]), as well as Thai (521 ng × h/mL [21]) and Indian (450 ng × h/mL [23]) patients with vivax malaria. This could be related to the increased sensitivity of mass spectrometry in the current analysis. Other key PK parameters, notably, the median/mean *T*_max_, t_1/2_, CL/*F*, and Vz/F of both formulations were also consistent with studies dating as far back as the 1980s [5–7, 15, 19–22]. Taken together, these previously published data support the validity of our PK findings. The inactive carboxyPQ metabolite was also measured for data completeness but readers should be aware this is not required for WHO prequalification.

The study’s main limitation was that women could not be recruited because they did not wish to join this study. Male female differences in PQ disposition remain to be resolved with a limited number of small studies reporting conflicting results; two [16, 24] report no sex differences whereas a Thai study reported higher exposure in 4 females vs. 4 males [25]. It is highly unlikely that female volunteers would show a systematic difference between the IPCA and Sanofi formulations, that was not demonstrated in the male volunteers. Methaemoglobinaemia, a known dose-related side effect of primaquine, was not measured. However, at this low dose, methaemoglobin levels would be expected to be low and not clinically relevant [26]. Finally, CYP2D6 was not genotyped to determine metabolizer status and its relationship to PQ and carboxyPQ exposure. A more rapid metabolizer status results in a greater concentration of the active oxidative metabolites (which are challenging to measure) and a lower exposure of PQ [27]. All of these limitations had no impact on the study’s primary endpoint of determining bioequivalence.

IPCA is one of several companies that have embarked on either prequalifying or registering PQ. Macleods in India prequalified their coated 15 mg tablet in December last year (2023) and the Medicines for Malaria Venture is supporting Fosun, a Chinese company, to develop 2.5 and 5 mg dispersible tablets of PQ [28]. A consortium, led by this paper’s first and senior authors, is also planning to prequalify a line of flavoured paediatric PQ tablets (2.5, 5 and 7.5 mg) by showing bioequivalence of a flavoured 15 mg tablet in adults and then obtaining a biowaiver on the basis of proportionality of the lower tablet strengths compared to the 15 mg tablet [29, 30].

Malaria endemic countries need PQ as an essential tool for malaria elimination to fill the substantial gap left by tafenoquine’s restricted licence of use in G6PD normal and minimally G6PDd deficient vivax-infected patients [31] and for blocking the transmission of artemisinin-resistant *P. falciparum* [1], which is well established in SE Asia [32] and is now emerging independently as a new threat in large parts of eastern Africa [33–36].

Cost is a critical issue because many countries depend on the Global Fund for purchasing large drug supplies but donor money may not always be available and then countries will need to pay for anti-malarials themselves. Data from the Global Fund show that the prices paid for the bulk purchase of PQ were 0.46 (2016) and 0.51 USD (2015) for one tablet of 15 mg of Valeant (Sanofi) made PQ, and 0.038 USD for one tablet of 7.5 mg made by Remedica [37]. The UNICEF web site offers 1000 7.5 mg PQ tablets (brand not mentioned but, presumably, Remedica) for 31.14 USD [38]. In Thailand, one bottle (250 tablets) of locally-manufactured PQ (Government Pharmaceutical Organization, GPO) costs 175 THB for a unit cost of ~ 0.02 USD/tablet (Kaendiao, personal communication). Therefore, a 14-day course of 15 mg/day of PQ for *P*. *vivax* radical cure would cost approximately 30 US cents (GPO) and 1 and 6.4 USD for Remedica and Sanofi, respectively. If a customized blister pack were to be used to aid adherence [39], then this cost would also need to be considered as well as the additional cost of shipping.

## Conclusions

The current market for PQ is huge, due mostly to the burden of *P. falciparum*, and will remain so in the foreseeable future. Single dose tafenoquine is likely to dominate the *P. vivax* market, if reliable and affordable G6PD testing can be assured on a large scale. There is room for several manufacturers and increased competition should further reduce the price of PQ. This study has shown bioequivalence of a new 15 mg tablet of generic PQ and the IPCA dossier is under review for WHO prequalification, and, if granted, registration in malaria endemic countries will follow. The primary use of IPCA’s 15 mg tablet will be in adults and adolescents for radical cure and transmission blocking.

### Supplementary Information

**Additional file 1: Table S1.** Laboratory reference values for an Indian population (Cliantha internal document).

## Data Availability

Selected data generated and analysed during this study are included in this published article. Deidentified individual participant data will be available to applicants who provide a sound proposal to the Mahidol Oxford Tropical Medicine Research Unit Data Access Committee. Such applicants should be aware that the prequalification of primaquine is current and so there will be restrictions on what data and reports we can provide before prequalification is granted. The senior author should be contacted in the first instance (bob@tropmedres.ac).

## Abbreviations

ACT: Artemisinin based combination treatment
ALT: Alanine transferase
AUC: Area under the drug concentration time curve
BE: Bioequivalence
BMI: Body mass index
CL: Total drug clearance from plasma
*C*_max_: Maximum drug concentration
EMA: European Medicines Agency
*F*: Drug bioavailability
FDA: Federal Drug Administration
G6PDd: Glucose-6-phosphate dehydrogenase deficiency
GM: Geometric mean
GPO: Government Pharmaceutical Organization
HIV: Human immunodeficiency virus
HPLC: High performance liquid chromatography
Ln: Natural log
MedDRA: Medical Dictionary for Regulatory Activities
PK: Pharmacokinetics
PQ: Primaquine
SRA: Stringent regulatory authority
TB: Tuberculosis
t_1/2_: Half-life
*T*_max_: Time to maximum drug concentration
UNICEF: United Nations International Children’s Emergency Fund
VDRL: Venereal disease reference laboratory
Vz: Volume of distribution based on the terminal elimination phase
