# Supplementary material for: Bioequivalence of a new coated 15 mg primaquine formulation for malaria elimination
Source: Malar J. 2024 Jun 5;23:176. doi: 10.1186/s12936-024-04947-6 (PMC11155120; doi:10.1186/s12936-024-04947-6)
Supplement: Supplementary file 1 — Additional file 1: Table S1. Laboratory reference values for an Indian population (Cliantha internal document). [file 12936_2024_4947_MOESM1_ESM.pdf]

cliantha research	**CLIANTHA RESEARCH**<br>**BIOLOGICAL REFERENCE INTERVAL**

Biochemistry

Test Name	Unit	Male	Female
Blood Urea Nitrogen (BUN)	mg/dL	6.00 – 20.00	6.00 – 20.00
Creatinine	mg/dL	0.60 – 1.20	0.60 – 1.20
Glucose – Random (RBS)	mg/dL	70.00 – 140.00	70.00 – 140.00
Fasting Glucose	mg/dL	70.00 – 110.00	70.00 – 110.00
Post Prandial Blood Sugar (PPBS)	mg/dL	< 140.00	< 140.00
Alkaline Phosphatase	U/L	20.00 – 130.00	20.00 – 130.00
Bilirubin - Total	mg/dL	0.19 – 1.32	0.19 – 1.32
Total Protein	g/dL	6.70 – 8.60	6.70 – 8.60
Albumin	g/dL	3.50 – 5.50	3.50 – 5.50
Sodium	mmol/L	136.00 – 145.00	136.00 – 145.00
Potassium	mmol/L	3.50 – 5.20	3.50 – 5.20
Chloride	mmol/L	96.00 – 106.00	96.00 – 106.00
Calcium	mg/dL	8.80 – 10.40	8.80 – 10.40
Phosphorus	mg/dL	2.70 – 4.50	2.70 – 4.50
Uric Acid	mg/dL	2.50 – 8.00	1.30 – 6.00
ALT (SGPT)	U/L	10.85 – 71.27	10.85 – 71.27
AST (SGOT)	U/L	14.79 – 48.68	14.79 – 48.68
Triglyceride	mg/dL	30.00 – 200.00	30.00 – 200.00
Total Cholesterol	mg/dL	Desirable : < 200.00<br>Borderline High: 200.00 – 239.00<br>High : ≥ 240.00	Desirable : < 200.00<br>Borderline High: 200.00 – 239.00<br>High : ≥ 240.00

**Hematology**			
**Test Name**	**Unit**	**Male**	**Female**
Hemoglobin	g/dL	12.0 – 16.5	11.0 – 15.0
Total RBC count	10^12/L	4.10 – 6.30	3.25 – 5.83
Total WBC count	10^6/L (/uL)	4432 - 11172	4432 - 11172
Platelet Count	10^6/L (/uL)	150000 – 410000	150000 – 410000
Hematocrit (HCT/ PCV)	%	40.0 – 50.0	36.0 – 46.0
Neutrophils	%	40.4 – 73.7	40.4 – 73.7
Lymphocytes	%	16.0 – 45.2	16.0 – 45.2
Monocyte	%	4.5 – 10.3	4.5 – 10.3
Eosinophils	%	0.6 – 10.4	0.6 – 10.4
Basophil	%	0.0 – 0.8	0.0 – 0.8
Absolute Neutrophil Count	/uL	2046 - 7050	2046 - 7050
Absolute Lymphocyte Count	/uL	1194 - 3878	1194 - 3878
Absolute Eosinophil Count	/uL	40 - 890	40 - 890
Absolute Monocyte Count	/uL	286 - 726	286 - 726
Absolute Basophil Count	/uL	0 - 50	0 - 50
Malarial Parasite	-	Absent	Absent

Urine Examination			
**Test Name**	**Unit**	**Male**	**Female**
pH (Reaction)	-	4.8 – 7.4	4.8 – 7.4
Specific Gravity	-	1.001 – 1.035	1.001 – 1.035
Albumin (Protein)	-	Negative	Negative
Sugar (Glucose)	-	Negative	Negative
Bilirubin	-	Negative	Negative
Urobilinogen	-	Negative	Negative
Ketones	-	Negative	Negative
Amorphous Material	-	Absent	Absent
Bacteria	-	Absent	Absent
Trichomonas	-	Absent	Absent
Monilia	-	Absent	Absent
Pus Cells	/HPF	0 – 3	0 – 3
Red Blood Cells	/HPF	0 – 3	0 – 3
Epithelial Cells	/HPF	0 – 3	0 – 5
Casts	/LPF	Absent	Absent
Crystals	-	Absent	Absent

Immunology / Serology			
**Test Name**	**Unit**	**Male**	**Female**
Anti-HCV	-	Non Reactive	Non Reactive
HIV I & II	-	Non Reactive	Non Reactive
HBsAg	-	Non Reactive	Non Reactive
Syphilis Test (Rapid Plasma Reagin)	-	Non Reactive	Non Reactive
β-Human Chorionic Gonadotropin (β-HCG)	IU/L	-	Non Pregnant Female: < 5.00

Approved by: Shaifali 12 Jul 21

Dr. Shaifali Gupta
(Laboratory Director)
